# Sulfur Induces Resistance against Canker Caused by *Pseudomonas syringae* pv. *actinidae* via Phenolic Components Increase and Morphological Structure Modification in the Kiwifruit Stems

**DOI:** 10.3390/ijms222212185

**Published:** 2021-11-10

**Authors:** Guifei Gu, Sen Yang, Xianhui Yin, Youhua Long, Yue Ma, Rongyu Li, Guoli Wang

**Affiliations:** 1 Engineering and Technology Research Center of Kiwifruit, Guizhou University, Guiyang 550025, China; 18798007047@163.com (G.G.); yangsen2008812@163.com (S.Y.); my00387812@163.com (Y.M.); ryli@gzu.edu.cn (R.L.); 2 Institute of Crop Protection, Guizhou University, Guiyang 550025, China; 3 Fruit Industry Development Service Centre, Guiyang 550200, China; yuanzhe866853@163.com

**Keywords:** sulfur, induced resistance, phenolic components, morphological structure, *Pseudomonas syringae* pv. *actinidiae*

## Abstract

Bacterial canker caused by *Pseudomonas syringae* pv. *actinidiae* (Psa) has led to considerable losses in all major kiwifruit-growing areas. There are no commercial products in the market to effectively control this disease. Therefore, the defense resistance of host plants is a prospective option. In our previous study, sulfur could improve the resistance of kiwifruit to Psa infection. However, the mechanisms of inducing resistance remain largely unclear. In this study, disease severity and protection efficiency were tested after applying sulfur, with different concentrations in the field. The results indicated that sulfur could reduce the disease index by 30.26 and 31.6 and recorded high protection efficiency of 76.67% and 77.00% after one and two years, respectively, when the concentration of induction treatments was 2.0 kg/m^3^. Ultrastructural changes in kiwifruit stems after induction were demonstrated by scanning electron microscopy (SEM) and transmission electron microscopy (TEM), and the activities of phenylalanine ammonia-lyase (PAL), peroxidase (POD) and polyphenol oxidase (PPO), and the accumulation of lignin were determined by biochemical analyses. Our results showed that the morphological characteristics of trichomes and lenticels of kiwifruit stem were in the best defensive state respectively when the sulfur concentration was 3.0 kg/m^3^ and 1.5 kg/m^3^. Meanwhile, in the range of 0.5 to 2.0 kg/m^3^, the sulfur could promote the chloroplast and mitochondria of kiwifruit stems infected with Psa to gradually return to health status, increasing the thickness of the cell wall. In addition, sulfur increased the activities of PAL, POD and PPO, and promoted the accumulation of lignin in kiwifruit stems. Moreover, the sulfur protection efficiency was positively correlated with PPO activity (*p* < 0.05) and lignin content (*p* < 0.01), which revealed that the synergistic effect of protective enzyme activity and the phenolic metabolism pathway was the physiological effect of sulfur-induced kiwifruit resistance to Psa. This evidence highlights the importance of lignin content in kiwifruit stems as a defense mechanism in sulfur-induced resistance. These results suggest that sulfur enhances kiwifruit canker resistance via an increase in phenolic components and morphology structure modification in the kiwifruit stems. Therefore, this study could provide insights into sulfur to control kiwifruit canker caused by Psa.

## 1. Introduction

Bacterial canker caused by *Pseudomonas syringae* pv. *actinidiae* (Psa) is a destructive global disease, and has become the primary problem in developing the kiwifruit industry worldwide [1,2,3]. Bacterial canker seriously affects the yields and quality of kiwifruit. There is still a lack of effective chemicals to prevent and control kiwifruit canker, and the irrational use of chemical pesticides threatens the quality and safety of its products. Therefore, it is crucial to seek new and effective methods to control cankers caused by Psa. Plant-induced disease resistance was considered to be a promising treatment as a substitute for the application of the chemical fungicide, which refers to the use of exogenous factors, including physical, chemical and biological factors, to pre-treat plants and induce their defense mechanism, so that the initial susceptible reaction produces local or systematic resistance [4,5,6]. Under the induction of exogenous substances, the structural, physiological and biochemical resistance of plants would change to a certain extent, such as the formation of papillae, lignification, the precipitation of callose, and the accumulation of phenolic compounds and disease-related proteins, which are closely related to plant resistance [7,8,9]. Studies on induced resistance have achieved good results in soybean [10], wheat [11], and melon [12,13], among others. The resistance induction of kiwifruit is considered to be a promising alternative method for controlling bacterial canker caused by Psa.

Sulfur is an essential element for the growth and development of plants. An appropriate sulfur content can improve yields and quality to some extent [14]. Studies have shown that plants are susceptible to diseases when they are sulfur deficient. When sulfur fertilizer is applied to the soil to stimulate sulfur-related metabolism, it can reduce the infection of pathogenic bacteria or restrain the spread of symptoms [15,16]. Bloem et al. and Haneklaus et al. proposed that sulfur, in addition to having a generally beneficial effect on the plant, which indirectly increases its ability to defend against pathogens, also acts by directly triggering the specific resistance response to pathogens [17,18]. In their study, Klikocka et al. found that sulfur fertilizer could improve the resistance of potato tubers to *Rhizoctonia solani* [16]. In addition, some studies have found that sulfur application in roots could increase the content of total phenols and lignin in tomatoes and the activity of related defense enzymes, and enhance the resistance of tomatoes to *Verticillium dahliae* [19,20], which further proved the existence of sulfur-induced resistance (SIR). Our previous research found that the toxicity of sulfur to kiwifruit canker in vitro was very weak, and the EC_50_ was only 1326.99 mg/L. However, when applied in the field, sulfur could reduce the incidence of kiwifruit canker, promote the maintenance of leaf cells and chloroplast structure, and significantly improve the quality of kiwifruit [21,22]. On the basis of verifying the field control effect, this experiment studied the morphological structure and physiological changes in kiwifruit after applying different concentrations of sulfur and organic fertilizer in soil, in order to reveal the mechanism of sulfur-induced kiwifruit resistance, and provide a basis for using fertilizers with appropriate sulfur content to control kiwifruit canker.

## 2. Results

### 2.1. Disease Severity and Protection Percentage after Sulfur Application

**Figure 1:**
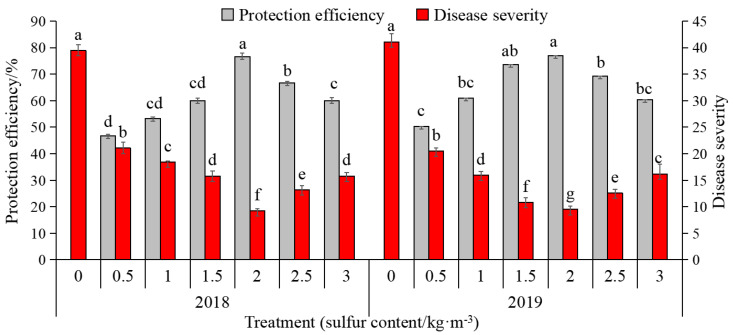
The effect of sulfur content on the disease index and protection efficiency of Psa_infected kiwifruit plants in fields. Vertical bars represented means (*n* = 3) ± standard deviations (SD). Different lowercase letters indicate significant differences between different treatments (*p <* 0.05). The same is shown below.

The means of disease severity and the relative protection efficiency, obtained in field trials, are shown in Figure 1. All tested sulfur concentrations applied individually significantly reduced the disease severity and increased protection efficiency, differing statistically from the non-treatments. It is worth mentioning that 2.0 kg/m^3^ is the best concentration to reduce the disease severity by 30.26 and 31.6, in 2018 and 2019, respectively. In addition, it exhibited a relatively high protection efficiency of 76.67% and 77.00% in 2018 and 2019, respectively. On the whole, compared with the incidence of kiwifruit canker in 2018, the disease index of kiwifruit canker without sulfur treatment in 2019 was aggravated, while the disease index of other sulfur treatments decreased and protection efficiency was improved, except for 0.5 kg/m^3^. The results showed that proper sulfur contents could improve the disease resistance of kiwifruit and reduce the severity of bacterial canker caused by Psa.

### 2.2. Changes in Morphological Structure of Kiwifruit Stem after Sulfur Application

#### 2.2.1. External Morphological Structure of the Stem

**Figure 2:**
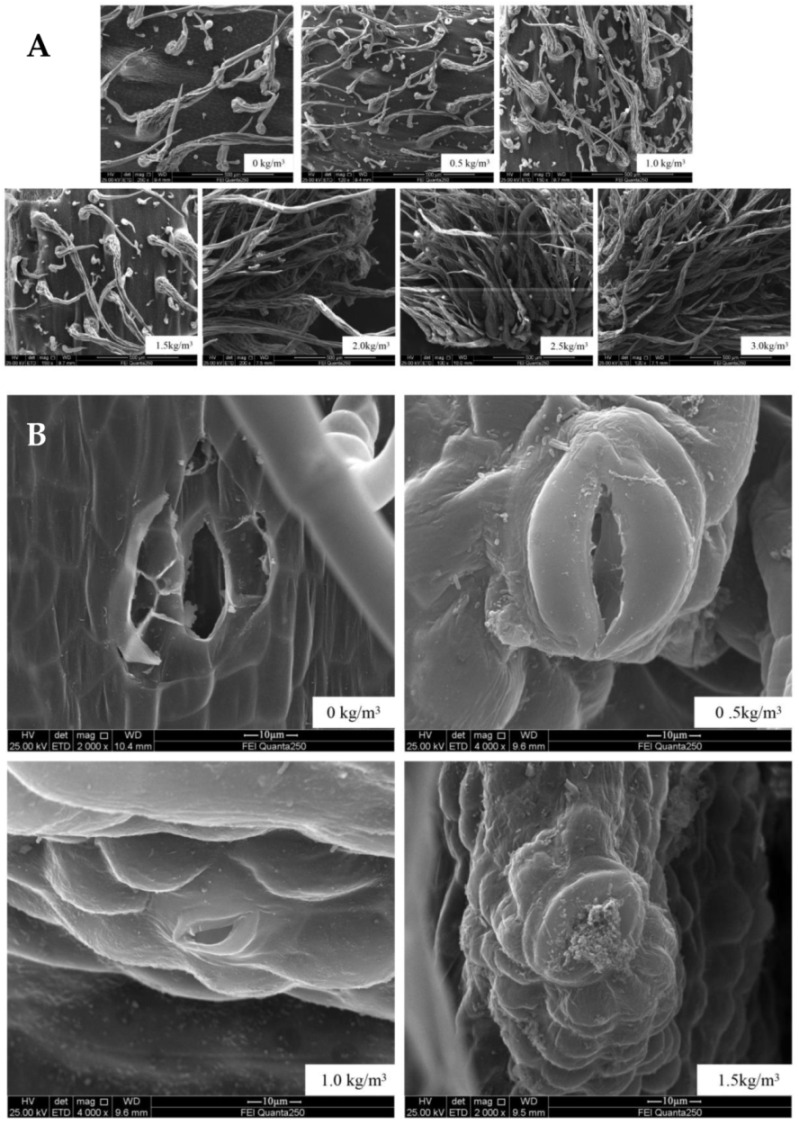
The distribution characteristics of trichomes in kiwifruit stems when sulfur contents are 0 to 3.0 kg/m^3^ (**A**), and the morphology of lenticels in kiwifruit stems when sulfur content is 0.5 to 1.5 kg/m^3^ (**B**).

Trichome: The effects of different sulfur contents on the distribution characteristics of trichomes in kiwifruit stems (Figure 2A). There was a tremendous difference in the morphology and quantity of kiwifruit stem trichomes between the sulfur treatment and control. After sulfur treatment, with the increase in sulfur concentration, the density of kiwifruit stem trichomes increased and lengthened. Among them, the distribution density of trichomes began to increase when the sulfur concentration was 0.5 kg/m^3^, and the density and length of trichomes increased and became longer compared to the control when the sulfur concentration was 1.0~2.0 kg/m^3^. Furthermore, the length and quantity of trichomes increased obviously, and the density distribution was dense when the sulfur concentration increased to 2.5 and 3.0 kg/m^3^. However, in the non-treatment group, there were fewer kiwifruit stem trichomes, which were few in number, sparsely distributed, and short in length. This indicated that suitable sulfur fertilizer could promote the growth of the kiwifruit stem trichomes.

Lenticels: As shown in Figure 2B, the lenticels of kiwifruit stem without sulfur treatment are single-thin-walled, and have large openings. When the sulfur content was 0.5 kg/m^3^, the lenticels of kiwifruit stems were thick, smooth, and elliptical. When the sulfur content was 1.0 to 1.5 kg/m^3^, the opening of the lenticels in kiwifruit stem became smaller. There was secretion at the opening, the lenticels were tight, and the surrounding tissues were stacked and distributed compactly, indicating that, when sulfur content was 1.5 kg/m^3^, the lenticels of kiwifruit stems were in a better defensive state.

#### 2.2.2. Ultrastructure of Stems

**Figure 3:**
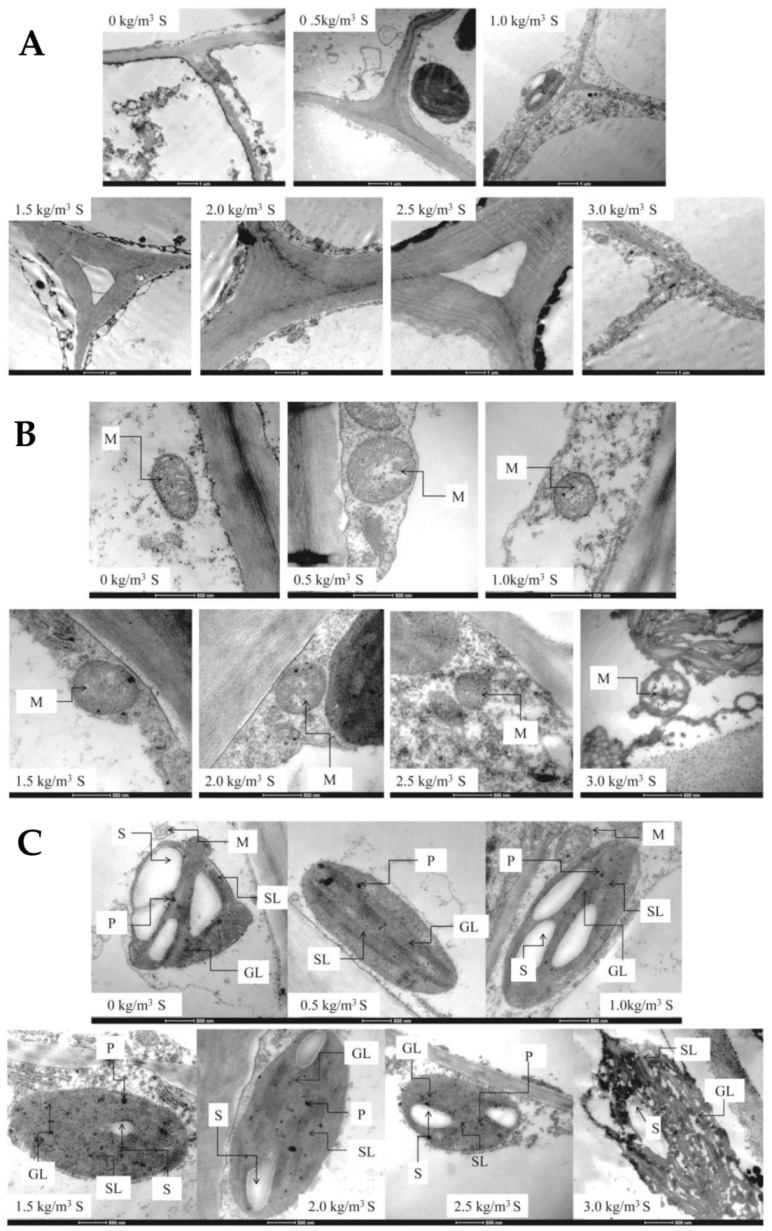
The effect of sulfur content on the ultrastructural morphology of kiwifruit stems where letters represent cytoderm (**A**) chloroplast ((**B**) M. mitochondrion; S. starch; GL. granum lamella; SL. stroma lamella; P. plastoglobulis.) and mitochondria ((**C**) M: mitochondria).

Cell wall: The effect of sulfur on the ultrastructure of the kiwifruit cell wall is shown in Figure 3A. Without sulfur treatment, the cell wall was thin, and the mitochondrial volume in tissues was small. In the range of 0.5 to 2.0 kg/m^3^, with the increase in sulfur content, the cell wall of the kiwifruit stems gradually thickened. After sulfur treatment at 2.0 kg/m^3^, the cell wall thickness reached the maximum, the shape was the most regular, and the color was more transparent. When the concentration of sulfur was more than 2.0 kg/m^3^, the effect of sulfur on the cell wall of the kiwifruit stems was unfavorable, the cell wall thickness decreased, and the tissue distribution was sparse and irregular. Therefore, the sulfur content can repair the cell wall of the kiwifruit stem that is susceptible to canker.

Chloroplast: As shown in Figure 3B. In the area seriously affected by kiwifruit canker, the chloroplast structure of the kiwifruit stems without sulfur treatment was seriously deformed and oval, its starch granule size was moderate, and its mitochondrial structure was clear, but the uniformity and transparency of the granules and matrix sheet were poor. Compared with the control, when the sulfur concentration was 0.5 kg/m^3^, the chloroplast matrix and grana lamellae were clear, and the shape was shuttle-shaped. When the sulfur concentration was 1.0 kg/m^3^, the chloroplast was shuttle-shaped, and the number and volume of starch granules increased obviously. When the sulfur concentration was 1.5 and 2.0 kg/m^3^, the number of osmiophiles increased obviously, and chloroplasts were distributed close to the cell wall. However, when the sulfur content was between 2.5 and 3.0 kg/m^3^, the shape of the chloroplast gradually became oval, the volume of the chloroplast and starch granules became smaller and began to dissolve, the matrix and granule gradually became blurred, and the color became darker, far away from the cell wall.

Mitochondria: The effect of sulfur on the mitochondrial morphology of kiwifruit stem is shown in Figure 3C. Without sulfur treatment, the mitochondria of stems were spindle-shaped, far away from the cell wall. After sulfur treatment, the shape, volume and distance from the cell wall of stem cell mitochondria changed to a certain extent. When sulfur treatment was 1.5 kg/m^3^, mitochondria were oval and distributed near to the cell wall, and osmiophilic particles increased. When the sulfur concentration was 2.0 to 3.0 kg/m^3^, the mitochondrial volume began to decrease, and its distribution began to move away from the cell wall. When the sulfur concentration was 3.0 kg/m^3^, the mitochondrion dissolved and split, with an irregular shape and a deeper color.

### 2.3. Effects of Sulfur Treatment on Phenolic Components in Kiwifruit Stems

#### 2.3.1. The Activities of POD, PPO, and PAL

**Figure 4:**
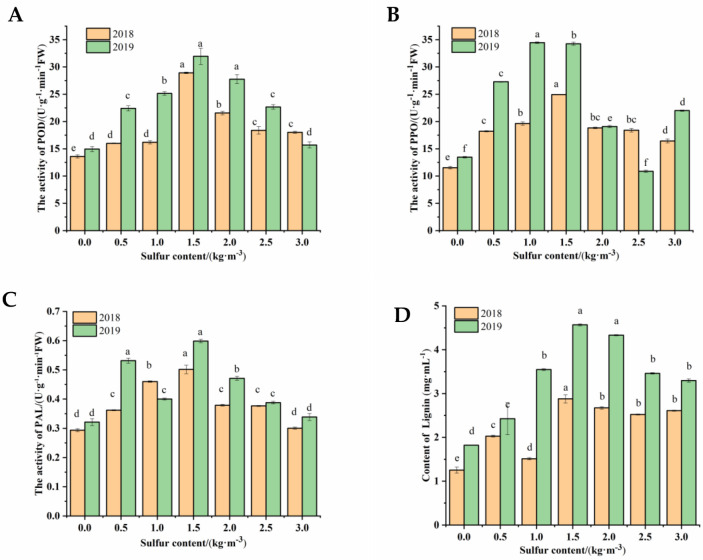
Effect of sulfur treatment on phenolic components of kiwifruit stems. (**A**) (POD activity), (**B**) (PPO activity), (**C**) (PAL activity) and (**D**) (lignin content) of Psa_infected kiwifruit stems. Different lowercase letters indicate significant differences between different treatments (*p <* 0.05).

As shown in Figure 4A, after sulfur treatment for one and two years, when the sulfur concentration was 0.5 to 1.5 kg/m^3^, the POD activity in kiwifruit stems increased significantly, reaching the maximum values of 28.93 and 31.95 U·g^−1^·min^−1^·FW (*p* < 0.01) at 1.5 kg/m^3^, respectively. Then, the activity gradually decreased until the sulfur content was 3.0 kg/m^3^, and there was no apparent difference in comparison with the control.

Compared to the control group, the PPO activity of sulfur-treated kiwifruit stems increased significantly after one and two years at a proper sulfur content (Figure 4B). In the range of 0.5 to 1.5 kg/m^3^, PPO activity gradually increased with the increase in sulfur concentration, and the highest value was 24.94 U·g^−1^·min^−1^·FW in 2018 and 34.26 U·g^−1^·min^−1^·FW in 2019 with the treatment of 1.5 kg/m^3^, which was 116.31% and 154.53% higher than those without sulfur treatment (*p* < 0.01). Then, the activities decreased slightly at 2.0 to 3.0 kg/m^3^.

PAL activity in sulfur-treated kiwifruit stems was significantly (*p* < 0.01) enhanced with 0.5 to 1.5 kg/m^3^ (Figure 4C). The activity increased sharply and reached its peak when sulfur content was 1.5 kg/m^3^. The activity was 70.66% in 2018 and 84.37% in 2019, which was higher than the control kiwifruit stem. Meanwhile, for other treatments, a decrease in activity was observed.

#### 2.3.2. Content of Lignin

The effect of sulfur on lignin content in kiwifruit stem is shown in Figure 4D. After sulfur treatment, the lignin content in kiwifruit stem increased compared with that of untreated kiwifruit. The lignin content of 1.5 kg/m^3^ sulfur treatment was the highest. It was 2.88 mg/mL in 2018 and 4.57 mg/mL in 2019, which were 130.40% and 150.10% higher than that of no sulfur treatment, respectively.

### 2.4. Correlation Analysis between Protection Efficiency and Phenolic Components in Kiwifruit Stems

After sulfur application for two years, the correlation between phenolic compounds in kiwifruit stems and protection efficiency was analyzed (Table 1). Protection efficiency had a positive correlation with PPO, POD and PAL activity and lignin content, among which PPO activity had a significant positive correlation (*p* < 0.05) and lignin content had a highly significant positive correlation (*p* < 0.01). In addition, PPO, POD, PAL and lignin are also positively correlated with one another. The results indicated that the protection efficiency was closely related to the content of phenols, especially the content of lignin.

## 3. Discussion

Sulfur is a structural component of life substance, and it participates in the photosynthesis, respiration and nitrogen metabolism of plants. The rational application of sulfur fertilizer not only increases the yield of plants and improves the quality of agricultural products, but also enhances the disease resistance of plants, which is of great significance to the whole normal process of crops [23,24]. Rossini et al. found that sulfur fertilizer could improve grain yield and quality [25]. The research of Ibaez et al. showed that sulfur deficiency had a significant effect on the growth process of soybean, and appropriate sulfur application could increase the yield of soybean and increase the storage amount of protein in grain [26]. Singh et al. found that a certain amount of sulfur and zinc could reduce the disease index of lentil powdery mildew [27]. Pavlista reported that the proper application of sulfur fertilizer could minimize the occurrence of potato scabs [28]. The results showed that sulfur had a specific protection efficiency against kiwifruit canker. After two years of sulfur application in the field, the incidence of kiwifruit canker decreased obviously. The protection efficiency was the best when the sulfur application was 2.0 kg/m^3^, while when the sulfur application was higher than 2.0 kg/m^3^, the protection efficiency on kiwifruit canker was poor; this may be due to the high sulfur content and low acidity in soil, which were not suitable for kiwifruit plant growth. The weak growth potential of kiwifruit resulted in the weakening of the kiwifruit’s resistance to canker. In this study, it was found that the sulfur application of 1.5 kg/m^3^ could improve the morphological characteristics of the lenticels of kiwifruit stems infected with Psa, make the lenticels tight in the surrounding overlapping tissues, and increase the surrounding secretions. At the same time, the density of trichomes on kiwifruit stems was positively correlated with the increase in sulfur content, which indicated that sulfur fertilizer could promote the growth of stem trichomes. The study also showed that the opening of kiwifruit lenticels and their secretion and trichomes had an excellent defense function against the invasion of pathogen of kiwifruit canker. From the ultrastructural point of view of kiwifruit stem, sulfur application in the concentration of 0.5 to 2.0 kg/m^3^ can repair the chloroplast, mitochondria and cell wall of infected kiwifruit stem, and gradually restore the shape of the chloroplast and mitochondria to spindle shape increase the number of starch granules in both organelles, making organelles closer to the cell wall, and increasing the cell wall thickness. The research results were similar to the conclusions of Yin et al. [22]. When the concentration of sulfur continuously increased to 2.5 or 3.0 kg/m^3^, the chloroplast, mitochondrial structure and cell wall thickness of the kiwifruit stem began to crack or dissolve and become thinner, which indicated that a high concentration of sulfur had certain adverse effects on kiwifruit photosynthesis. The results were similar to those reported by Ostaszewska et al. [29]. Therefore, an appropriate amount of sulfur can change the morphological characteristics of kiwifruit stem organelles, prevent the occurrence of organelle dissolution, promote the recovery of cracked organelles, and increase the number and volume of starch granules and cell wall thickness in organelles, which may be an important physiological effect of sulfur-induced kiwifruit resistance to canker.

Phenolic components include lignin, soluble phenolic compounds, phenolic phytochemicals, and other antibacterial substances, all of which are metabolites of phenylpropane. The accumulation of phenolic components in plants is often part of the defense response against the attack and stress of pathogens, which can be triggered and activated by elicitors [30]. Deenamo et al. found that salicylic acid could induce resistance in rubber tree against *Phytophthora palmivora* by promoting the activities of H_2_O_2_, catalase (CAT), POD and PAL, increasing the content of lignin [31]. Similar findings have been found in other plant disease resistance studies [32,33]. This study showed that the application of sulfur with a concentration of 0.5 to 2.0 kg/m^3^ could increase the activities of POD, PPO and PAL and lignin content in the kiwifruit stem, and enhance the kiwifruit’s resistance to bacterial canker. Based on the induced metabolic response of plants to endogenous protective defenses against biotic and abiotic stresses, the use of phenolic substances against bacterial pathogens is an exciting prospect. Furthermore, the biosynthesis of stress-induced secondary metabolites, such as antimicrobial phenols, is not only a part of the natural adaptive response of plants to pathogen attack, but also a part of reducing the harm caused by changes in environmental conditions and related abiotic stress factors [34]. Zhang et al. reported that exposure to various abiotic and biological stresses regulates the expression of genes encoding key enzymes in the phenol biosynthesis pathway, such as PAL [35]. It was also found that lignin content and PPO activity were significantly correlated with the control effect of sulfur application on kiwifruit bacterial canker. Defensive-induced lignification is a conserved basic defense mechanism in plant immune response to a variety of pathogens [36,37]. It has been reported that many enzymes are involved in the biosynthesis of lignin [35,38]. Therefore, it is speculated that the increase in lignin content in stem after sulfur treatment may be related to kiwifruit’s resistance to canker pathogen infection. However, to date, only the effect of sulfur-induced resistance to kiwifruit canker on lignin content has been studied, and the function of lignin in sulfur-induced resistance to kiwifruit canker can be further verified by cloning and gene editing techniques, thus determining the relationship between lignin and kiwifruit canker. At the same time, there may be various pathways of sulfur-induced disease resistance in plants, which is not limited to the above-mentioned aspects. Further molecular studies are needed to reveal the signal pathways and defense pathway of sulfur-induced resistance to bacterial canker in kiwifruit.

## 4. Materials and Methods

### 4.1. Kiwifruit Plants

The variety of kiwifruit is named ‘Hongyang’, which is 18 years old and can be planted at 74 plants/667 m^2^. This commercial cultivar was chosen as it is commonly used in the kiwifruit-growing areas of China for its good adaptation and high yields, and examined for the presence of Psa bacterial canker infection.

### 4.2. Fertilizers and Reagents

Refined organic fertilizer (total nutrients ≥ 4%, and organic matter ≥ 30%) and sulfur powder (sulfur content 95%) were provided by Guizhou Jilong Ecological Technology Co., Ltd. (Guiyang, China). 4% glutaraldehyde fixed solution was purchased from Qingdao Jieshikang Biotechnology Co., Ltd. (Qingdao, China). PBS buffer and enzyme activity determination kit were obtained from Beijing Baiaolaibo technology Co., Ltd. (Beijing, China).

### 4.3. Instruments and Equipment

T6 new century ultraviolet-visible spectrophotometer (Beijing, China), Scanning electron microscope (FEI Quanta 250, USA), Transmission electron microscope (JEM-2100, Japan), Ultramicrotome (Leica ultracut R, Germany).

### 4.4. Field Trials

The test was conducted in a Kiwifruit Plantation in Xiuwen county (26°55’57.81” N, 106°37’42” E), Guizhou Province, in December 2017. This is a kiwifruit orchard that was seriously infected by Psa, and the incidence rate reached 77.9% in 2017. According to the estimation of the crown size of kiwifruit, the root soil volume of each plant was calculated as 1 to 1.5 m^3^. The experiment was conducted in a randomized block design, with seven treatments (Table 2). There were three kiwifruit trees in each treatment. After sulfur powder and organic fertilizer were mixed evenly, each plant was applied as a base fertilizer using the ring ditch method, which was repeated 4 times, and a protective row was arranged between each treatment. During the experiment, the management level of each treatment was the same, and the field management was conducted according to the local kiwifruit cultivation techniques.

### 4.5. Investigation on the Occurrence of Disease

After sulfur treatment, the symptoms of the disease were assessed in April 2018 and April 2019. Furthermore, the disease index was evaluated with six grades: 1 (there were no disease spots), 2 (less than 1/3 of the branches were sick or wilted, or the pathological changes in the main stem did not exceed 1/3 of the stem circumference), 3 (1/3 to 1/2 branches became sick, withered or even died, or 1/3 to 1/2 of the stem circumference is surrounded by diseased spots of the main stems), 4 (1/2 to 3/4 of the branches died of disease or wilting, or 1/2 to 3/4 of the stems were surrounded by disease spots), 5 (more than 3/4 of the branches were diseased or wilted, or more than 3/4 of the stem circumference was surrounded by the disease spot of the main stem), 6 (the whole plant died). The disease severity and protection efficiency were calculated by the following formula.

Disease severity (DS) = ∑(Ni × i)/(N × 6) × 100;(1)

where Ni indicated the number of infected stems, and i indicated the representative value of this level, and N indicated the total number of investigated stems.

Protection efficiency (PE)% = (CK − DS)/CK × 100%;(2)

where CK indicated control average disease severity, and DS indicated treatment average disease severity.

### 4.6. Observation on the Morphological Structure of Stems

On 10 September 2019, the young kiwifruit stems were collected in the field experimental orchard, immediately fixed with glutaraldehyde, and then brought back to the laboratory to observe the sub-ultrastructure. Four stems were randomly taken from each treatment.

Lenticels and Trichome: The stems of the kiwifruit orchard were collected for cryopreservation and brought back to the laboratory for scanning electron microscopy. Ten fields of vision were selected for each sample. Samples were prepared as follows. Fixation: fixation with 4% glutaraldehyde for 2 to 4 h; cleaning: PBS (0.1 M/L pH: 7.2 to 7.4) was used three times, and each time lasted 1 min; drying: soak in hexamethyldisilazane for 3 min, take out the sample after soaking, and place it into a dryer for natural drying. After drying, a sputtering ion instrument (Platinum 30 s) was used, followed by observation using the scanning electron microscope (FEI Quanta 250, USA).

Ultrastructure: Selecting new young shoots of kiwifruit. Ten fields of vision were selected for each sample. Samples were prepared as follows. Pre-fixation: 1 cm × 1 cm tissue mass was cut and fixed with 4% glutaraldehyde for 2 to 4 h; cleaning: PBS (0.1 M/L pH: 7.2 to 7.4) was used 3 times, each time lasting 15 min; post-fixation: 1% osmium acid (0.24M PBS pH 7.4) was fixed for 2 h; Cleaning: PBS (0.1M/L pH: 7.2 to 7.4) was used 3 times, each time lasting for 15 minutes; gradient ethanol dehydration: 50%, 70%, 80%, 90%, 95% and 100% for 15 min each time, with acetone: ethanol = 1:1 (15 min), pure acetone (15 min/twice); infiltration: acetone: embedding agent = 2:1 (1 h), acetone: embedding agent = 1:1 (3 h), acetone: embedding agent = 1:2 (3 h), pure embedding agent (overnight); embedding and polymerization: 37 °C for 12 h, 45 °C for 12 h and 60 °C for 48 h. Epon812 embedding agent contains (10 g): Epon 812: 5 g, DDSA: 1.7 g, NMA: 3.5 g and DMP-30: 0.2 g. Then, the dried samples were cut into 70 nm thick slices with an ultrathin slicer, and then the slices were stained using the 3% uranium acetate lead citrate double staining method. Finally, cell walls, chloroplasts, and mitochondria were observed and photographed using a transmission electron microscope.

### 4.7. Determination of Phenolic Component Activity in Stems

The young stems were collected after sulfur treatment for 1 and 2 years, wrapped with tin foil paper, placed a 50 mL centrifuge tube, and stored in liquid nitrogen. The activities of phenylalanine ammonia-lyase (PAL), polyphenol oxidase (PPO) and peroxidase (POD) in kiwifruit stems were measured in the laboratory. All the methods were determined according to the kit of Solarbio Company. The methods of Bhaskara Reddy et al. [39] and Lee et al. [40] were used for the determination of lignin. Each treatment was repeated four times.

### 4.8. Statistical Analysis

Microsoft Excel 2010 (Microsoft Crop., Redmond, WA, USA) was used to sort the data, and SAS statistical software was used to analyze the data by one-way ANOVA. Tukey’s F test showed that there were significant differences among different treatments, with *p* < 0.05 being significant, and *p* < 0.01 being extremely significant.

## 5. Conclusions

When plants are infected with pathogens, sulfur has a high potential to induce disease resistance. In this study, sulfur was confirmed to be efficient in reducing canker caused by Psa, which infects kiwifruit plants following the assessment of field trials. Sulfur with a content of 2 kg/m^3^ reduced the disease severity by 30.26 and 31.6 separately in two years, and recorded a high protection efficiency of 76.67% in 2018 and 77.00% in 2019 compared to non-treated plants. Additionally, the lenticels, trichomes, chloroplasts, mitochondria and cell walls of kiwifruit stems showed obvious morphological modification after appropriate sulfur treatment. Kiwifruit plants treated with appropriate sulfur showed a significant increase in PAL, POD, PPO, and lignin content compared to the non-treated infected kiwifruit plant. In particular, sulfur protection efficiency had a highly significant positive correlation with lignin content, and was positively correlated with PPO activity. Thus, our results show that treatments in which sulfur presented resistance-inducing activity possibly mediated by the increase in the formation of phenolic components increase and morphological structure changes in stems contribute to the protection of kiwifruit plants against canker caused by Psa.

## Figures and Tables

**Table 1:** Correlation coefficient between phenolic components in kiwifruit stems and protection efficiency.

Correlation Coefficient	Protection Efficiency/%	POD Activity /U·g^−1^ min^−1^ FW	PPO Activity /U·g^−1^ min^−1^ FW	PAL Activity /U·g^−1^ min^−1^ FW	Lignin Content /mg·mL^−1^
Protection efficiency/%	1				
POD activity/U·g^−1^ min^−1^ FW	0.35	1			
PPO activity/U·g^−1^ min^−1^ FW	0.71 *	0.56	1		
PAL activity/U·g^−1^ min^−1^ FW	0.51	0.6	0.83 *	1	
Lignin content/mg·mL^−1^	0.88 **	0.39	0.81 *	0.52	1

Note: * *p* < 0.05; ** *p* < 0.01.

**Table 2:** The concentration of sulfur treatments.

Number	Treatment
S_0.5_	0.5 kg/m^3^ Sulfur powder * + 10 kg Organic fertilizer
S_1.0_	1.0 kg/m^3^ Sulfur powder + 10 kg Organic fertilizer
S_1.5_	1.5 kg/m^3^ Sulfur powder + 10 kg Organic fertilizer
S_2.0_	2.0 kg/m^3^ Sulfur powder + 10 kg Organic fertilizer
S_2.5_	2.5 kg/m^3^ Sulfur powder + 10 kg Organic fertilizer
S_3.0_	3 kg/m^3^ Sulfur powder + 10 kg Organic fertilizer
S_0_	10 kg Organic fertilizer, CK

* Note: The sulfur powder 0.5 kg was added to 1 m^3^ soil.

## Data Availability

The datasets generated or analyzed during the study are available from the corresponding author upon reasonable request.
